# Geopolymerization of Untreated Dredged Sediments for Sustainable Binder Development

**DOI:** 10.3390/ma19020433

**Published:** 2026-01-22

**Authors:** Lisa Monteiro, Humberto Yáñez-Godoy, Nadia Saiyouri, Jacqueline Saliba

**Affiliations:** 1 Arts et Metiers Institute of Technology, University of Bordeaux, Centre National de la Recherche Scientifique (CNRS), Bordeaux INP, I2M, UMR 5295, 33400 Talence, Francehumberto.yanez-godoy@u-bordeaux.fr (H.Y.-G.);; 2 College of Engineering and Technology, American University of the Middle East, Egaila 54200, Kuwait

**Keywords:** sediment valorization, sustainable materials, alkali activation, aluminosilicates, mechanical strength

## Abstract

The valorization of dredged sediments represents a major environmental and logistical challenge, particularly in the context of forthcoming regulations restricting their marine disposal. This study investigates the potential of untreated dredged sediments as sustainable raw materials for geopolymer binder development, with the dual objective of sustainable sediment management and reduction in cement-related environmental impact. Dredged sediments from the Grand Port Maritime de Bordeaux (GPMB) were activated with sodium hydroxide (NaOH) and sodium silicate (Na_2_SiO_3_), both alone and in combination, with supplementary aluminosilicate and calcium-rich co-products, to assess their reactivity and effect on binder performance. A multi-scale experimental approach combining mechanical testing, calorimetry, porosity analysis, Scanning Electron Microscopy and Energy-Dispersive Spectroscopy (SEM–EDS), X-ray diffraction (XRD), Thermogravimetric Analysis (TGA), and solid-state Nuclear Magnetic Resonance (NMR) was employed to challenge the commonly assumed inert behavior of sediments within geopolymer matrices, to elucidate gel formation mechanisms, and to optimize binder formulation. The results show that untreated sediments actively participate in alkali activation, reaching compressive strengths of up to 5.16 MPa at 90 days without thermal pre-treatment. Calcium-poor systems exhibited progressive long-term strength development associated with the formation of homogeneous aluminosilicate gels and refined microporosity, whereas calcium-rich systems showed higher early age strength but more limited long-term performance, linked to heterogeneous gel coexistence and increased total porosity. These findings provide direct evidence of the intrinsic reactivity of untreated dredged sediments and highlight the critical role of gel chemistry and calcium content in controlling long-term performance. The proposed approach offers a viable pathway for low-impact, on-site sediment valorization in civil engineering applications.

## 1. Introduction

With the upcoming French blue economy laws set to prohibit the discharge of polluted dredged sediments into the sea by January 2025, it is crucial to find ways to utilize these sediments and create a sustainable economy for managing them [1]. Traditionally, dredged sediments have been seen as waste, and, when disposing of them in the sea was not an option, they were used to fill quarries or strengthen banks [2,3]. Recent efforts have focused on recycling dredged materials in civil engineering to support a circular economy, reduce the use of non-renewable materials, and minimize the environmental impact [4]. Dredged sediments have the potential to serve as a valuable source of materials due to their large volume. Various research projects have shown the benefits of incorporating sediments into concrete, either by substituting sand or cement [2,5,6,7]. Although sand can be incorporated directly without any preliminary processing [8,9], no established method currently allows sediments to be used in cement production unless they undergo calcination or another form of chemical activation [10,11,12,13]. In this context, geopolymerization emerges as a promising alternative, enabling dredged sediments to function as a binder without requiring prior treatment. This approach offers a two-fold advantage: it addresses the management issues related to dredged sediments and reduces the environmental impact by cutting down on greenhouse gas emissions associated with using cement as the primary binder in construction. The term “geopolymer” was first introduced in 1978 by Joseph Davidovits [14] to describe inorganic polymers with a three-dimensional semi-crystalline aluminosilicate structure. This process is based on the reaction between an aluminosilicate material, called a precursor, and an alkali reagent to form a geopolymer. Davidovits [15] estimates that the manufacturing process requires nine times less energy than the production of Portland cement and emits eight to ten times less greenhouse gases. In addition to its energy benefits, geopolymerization allows the integration of aluminosilicate waste into the process. Much research is focused on the use of Fly Ash (FA), Ground-Granulated Blast Furnace Slag (GGBFS), and Metakaolin (MK) for geopolymerization. Although their viability as precursors has been proven, there is a growing trend to look beyond these three resources. Furthermore, Assi et al. [16] indicated that a significant number of industrial wastes conventionally used as precursors will not be sufficient to replace a significant part of the world’s production of Portland cement. Therefore, the primary challenge for developing more competitive geopolymers in the market will be to reduce environmental and economic costs by using locally available precursor sources as raw materials. The extension to natural aluminosilicate materials, such as marine dredged sediments, could be a relevant solution, especially as the clayey nature of the fine fraction of the sediments, which represents a barrier for their use in construction, is a real asset in geopolymerization.

**Figure 1:**
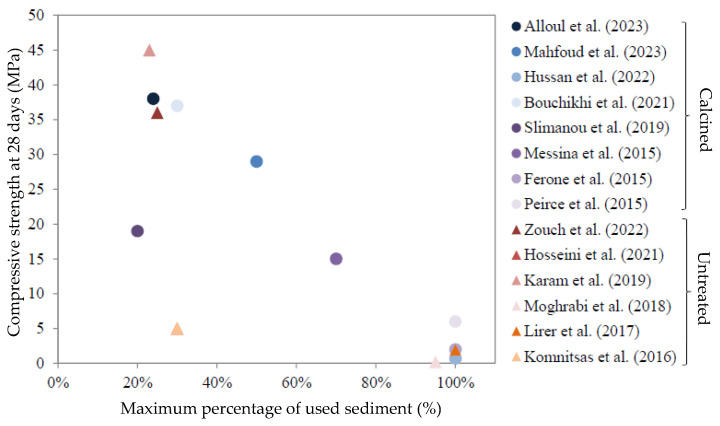
Compressive strength according to the maximum amount of sediment used in the literature [17,18,19,22,23,24,25,26,27,28,29,30,31,32,33].

The idea of finding a common solution to the environmental problems associated with two fundamental anthropogenic activities, such as sediment management and building material production, through geopolymerization has already been proposed in the literature [17,18,19,20]. Nevertheless, despite their suitable mineralogical compositions, few studies valorize dredged sediments in geopolymerization processes. In fact, their variable mineralogical properties, the existence of different phases (pollutants and organic matter, etc.), and their low reactivity make their use complex. In current studies, the use of dredged sediments for geopolymerization is performed either through the substitution of the precursor (FA, GGBFS, or MK), considering the sediment as inert, or by the calcination of sediments beforehand to modify their crystalline structure, thus improving their reactivity. Karam et al. [17] sought to alkali-activate a mixture of 77% GGBFS with 23% ground sediment using hydroxide and Na_2_SiO_3_ as alkaline activators. They observed that the addition of sediments decreased the compressive strength by almost 27% compared to a mixture with GGBFS alone. This decrease was attributed to the increase in porosity with the addition of sediment due to the dilution effect of the slag, the high total water demand of the sediment, and/or the antagonistic or synergistic effect of the constituent phases. They concluded that the sediments can neither be considered as inert elements nor as reactive in the alkaline activation as they affect the overall properties of the system. Ferone et al. [20] also observed the impact of adding sediments calcined at 400 °C to a mixture with GGBFS activated with 35% of Na_2_SiO_3_ solution and 10% of NaOH solution. The use of calcined sediments alone with silicate achieved a compressive strength of 4 MPa at 28 days compared to 32 MPa with the use of GGBFS. The study concluded that the addition of GGBFS and heat treatment of sediments is necessary to obtain a better degree of geopolymerization and, consequently, better mechanical performance. Hosseini et al. [19] valorized sediments extracted from a waterway in south-east Texas, treated mechanochemically with FA and Na_2_SiO_3_ to develop a geopolymeric mortar. When 25% and 50% of dredged sediment were added to the mix, compressive strength decreased by 17% and 54%, respectively. They concluded that the sediment reacts as an inert filler rather than as a precursor in the geopolymerization reaction. A similar conclusion was shared by Lirer et al. [18], who sought to activate a mixture of FA and marine dredged sediments for geopolymer synthesis. As the dredged sediment content increased, the mechanical properties of the mixtures in the study decreased, with a mixture composed of 100% sediment, until a compressive strength value of 2 MPa was reached. The addition of dredged sediments modifies the final structure of the geopolymeric matrix, which becomes less compact, indicating that the sediments do not play an active role in the geopolymerization process and act as an inert element. On the other hand, other studies consider sediments, not as inert materials, but as possible precursors to geopolymerization, provided that they are thermally or mechanically activated beforehand. This is the case of Mostefa et al. [21], who aims to determine the feasibility of using sediments from the Fergoug dam in Algeria to develop a geopolymerized binder. The sediments calcined at 750 °C are mixed with a NaOH solution at a ratio of liquid/solid = 0.8 with different molar ratios. Binders developed in this way can reach strengths ranging from 9 MPa to 14 MPa. The geopolymerization of calcined sediments from 650 °C to 750 °C has also been studied by Peirce et al. [22]. Highly alkaline solutions of sodium and potassium aluminate (8 M to 17 M) were used for the formation of geopolymers. The mechanical strengths of 5 MPa to 7 MPa obtained at 14 days for the potassium and sodium solution allowed the authors to conclude on the feasibility of using a calcined reservoir of dredged sediments in the development of construction materials. A summary of the sediment geopolymerization results in the literature is presented in Figure 1 [23].

As shown, few studies use dredged sediments for geopolymer formulation. In fact, the low compressive strengths obtained as their percentage in the geopolymerized matrix increases represent a barrier to their use. As can be seen in Figure 1, studies using 100% of their sediments achieve low compressive strengths despite the use of heat treatments to make the sediments more reactive. The variable mineralogical properties of the sediments, but also the existence of different phases (pollutants and organic matter, etc.), and their low reactivity make their use complex. Most studies to date, involving geopolymeric systems based on dredged sediments, have mainly focused on the use of heat treatment or the addition of supplementary materials to improve the compressive strength of the mixtures. These results are interesting from a mechanical point of view and allow for the co-use of sediments with other waste. However, a recent life cycle assessment conducted by Turner et al. [34] comparing the CO_2_ emissions generated by the manufacture of a cubic meter of Ordinary Portland Cement (OPC) concrete and with a geopolymer binder shows that the actual difference in carbon footprint between geopolymer binders and OPC binders is only 9%, which is much less than the values predicted by previous studies. The key factors that led to higher-than-expected emissions were the source of the raw materials, which can involve significant transport, the high alkali content in the material design, and the requirement for high-temperature processing of the geopolymer binder. These processes involve significant environmental and economic costs [35,36,37] and do not facilitate the reuse of sediments for on-site concrete directly. Thus, the main challenge for the development of more competitive geopolymers on the market will be to reduce costs by using locally available and untreated waste as the raw material source, to decrease the dosage of activator, and to improve the understanding and optimization of mixture formulation.

Despite the growing interest in sediment-based construction materials, most existing geopolymer studies either consider dredged sediments as inert fillers or rely on thermal or mechanical activation to enhance their reactivity. Such approaches increase energy demand, require centralized processing, and limit large-scale, on-site implementation. Moreover, the mechanisms governing reaction kinetics, gel formation, and long-term mechanical performance of sediment-rich geopolymer systems remain insufficiently understood, particularly under low-alkali concentrations and ambient curing conditions. In this context, the present study aims to investigate the intrinsic reactivity of untreated dredged sediments in alkaline environments, without prior calcination or chemical pre-treatment. The specific objectives are as follows: (i) to determine whether untreated sediments actively participate in geopolymerization or behave as inert components; (ii) to evaluate the influence of different co-products on reaction kinetics, gel chemistry, and mechanical performance; and (iii) to identify the dominant binding phases governing strength development and microstructural evolution. By combining mechanical, thermal, mineralogical, and spectroscopic analyses, this work establishes clear structure–property relationships and proposes a low-impact sediment valorization strategy suitable for practical civil engineering applications, particularly for public works in proximity to port infrastructures.

## 2. Experimental Methods and Materials

### 2.1. Dredged Sediments Characterization

#### 2.1.1. Origin and Dredging Method

The sediments used in this study were obtained from a dredging campaign conducted by the GPMB in March 2021. They were dredged in the Pauillac channel in the Gironde estuary [38]. The sediments were dredged using a suction pipe placed alongside the dredger and then stored in a well with a capacity of 3000 m^3^ before being discharged via a flap valve into the submerged areas. Sampling was carried out in the dredge well at several locations. After ensuring that enough was collected, the samples were packed in sealed plastic drums to ensure no interaction with the material. The samples were then homogenized to ensure that the material was representative, sieved to 2 mm to remove organic debris, and placed in bins with geotextiles. The initial water content of the Pauillac sediments was approximately 145%, expressed on a dry-mass basis. This high value is explained by the dredging and sampling method. As the choice of the study was not to thermally treat the sediments, natural drying was carried out through atmospheric evaporation and drainage caused by draining materials. This method was inspired by Allariz [39].

#### 2.1.2. Physical Characterization

The Garonne estuary is mainly composed of two distinct grain size fractions: sediments with strong sandy tendency upstream and silty–muddy sediments downstream. Since the objective here is to valorize the fine fraction of the sediments, silty–muddy sediments have been used. To determine the distribution of the grains constituting the sediments, an analysis was carried out using the Malvern Mastersizer 2000 laser (Malvern Panalytical, Malvern, UK) according to the standard NF P94 056 [40]. This analysis revealed a predominantly silty composition (67.8% to 73.6%) containing sandy particles (28.22% to 21.83%) with a small clay fraction (3.98% to 4.57%) [41]. It should be noted that the clay fraction considered here also constitutes the backbone of the polycondensation reaction [42]. Furthermore, a density value of 1.47 g/cm^3^ on average is observed, which is in line with the densities regularly observed in the Pauillac channel.

As the Pauillac sediments are fine materials, their sensitivity to water is high and can change their behavior. The Methylene Blue Value (MBV) analysis, carried out in accordance with the French standard NF P94 068 [43] and the Atterberg limits NF P94 051 [44], confirmed the silty-clay nature of the soil with a low plasticity. Water content was measured following XP P94 047 [45]. The organic matter content of the sediments was measured by loss on ignition at 550 °C based on standard NF EN 12879 [46]. The organic matter contents of the sample show low organic contamination. Table 1 summarizes the physical parameters of the Pauillac sediments.

#### 2.1.3. Chemical Characterization

X-ray diffraction (XRD) analysis is one of the fundamental techniques applied for the characterization of precursors for geopolymerization. It allows the identification of crystalline and amorphous phases to determine their dissolution potential in an alkaline solution [15]. This dissolution enables the production of silica and alumina oligomers suitable for polycondensation. The mineralogical characterization of the sediment was carried out both qualitatively and semi-quantitatively. Identification of the mineralogical phases was carried out using a Bragg–Brentano D8 A25 diffractometer with an angular range of 2θ = 5–90° and a step size of 0.02 °2θ. X-ray diffraction was also conducted to quantify and compare the crystal structures of the different formulations. The identification of the crystalline phases was performed using Profex 5.6.1 software (https://www.profex-xrd.org/, accessed on 3 January 2021) [47] with an accuracy of χ^2^ < 1.5. The XRD spectrum shows many reflection phenomena, suggesting the presence of many crystalline phases [41]. The analysis of the diffractogram obtained shows the presence mainly of the quartz and muscovite. The presence of minority phases, such as albite, kaolinite, and calcite, is also to be noted. The sediments contain two main phases: non-clay minerals, mainly quartz, and clay minerals, such as kaolinite. The proportion of amorphous phase in the sediments is 12.80%.

SEM tests were also conducted to qualitatively visualize the differences in microstructure and morphology. Energy-dispersive spectroscopy coupling was conducted to calculate the elemental compositions. The quantitative oxide composition of Pauillac sediments was obtained by SEM and is presented in Table 2. The predominant components are silica (Si) and alumina (Al), making the Pauillac sediments an aluminosilicate material. The sediments were also subjected to environmental tests in accordance with the decree of 9 August 2006 [48] and the GEODE guide [49], which determine the reference levels in France. No traces of heavy metal, PAH, and PCB contamination were observed. In fact, the contamination levels measured were low and below the N_1_ and N_2_ thresholds.

### 2.2. Synthesis of Sediment-Based Geopolymer

Nowadays, the geopolymerization reactivity of aluminosilicate precursors is defined as their degree of dissolution, precipitation, and polymerization in geopolymerization. However, there is a lack of standards to define criteria making aluminosilicate waste suitable for geopolymerization, leading to another barrier to the wide application of geopolymerization [50]. Herein, the feasibility of geopolymerization of soils is often linked to findings of studies rather than on existing standards. A fundamental requirement for using solid precursors in geopolymer synthesis is that they contain an amorphous or partially crystalline phase. This reactive fraction can dissolve in alkaline or acidic media, releasing Al- and Si-based oligomers that subsequently assemble into the polysialate network, forming the geopolymer gel. The crucial role of the amorphous aluminosilicate phase in raw materials has been highlighted by Singh et al. [51] and Wang et al. [52], where changes in the amorphous phase has been observed when using FA with alkaline reagent to form geopolymer mortars. Other factors, such as the mineralogy of clays and the content of Al and Si, allow a first evaluation of the potential of raw materials to be used as precursors. Marsh et al. [53] successfully produced geopolymer-stabilized soil without calcination using natural clay composed of high Si and Al content. Therefore, even if it is difficult to quantify the extent of phase transformations of aluminosilicate wastes, these criteria could indicate whether the sediments of the GPMB could be used as precursors for the formation of geopolymers.

Thus, the physico-chemical characterization of the Pauillac sediments shows their potential for use as precursors. Three parameters allow us to justify their use in the study. Firstly, the quantities of SiO_2_ and Al_2_O_3_, determined by EDS, in the Pauillac sediments had a ratio of SiO_2_/Al_2_O_3_ = 2.94. They were calculated by considering the global elemental composition of the sediments. The amorphous phase content was 13%. It is therefore concluded that the Pauillac sediments are suitable for use as a precursor.

#### 2.2.1. Alkali Reagent

In view of the bibliographical studies carried out, the alkali reagents selected in this study are the alkali reagents usually chosen in the geopolymer field. This choice allows us to reduce the scope of the study and compare the results with the literature. The alkali reagent solution used was prepared by mixing NaOH and Na_2_SiO_3_. The NaOH was used in the form of pellets at 97% purity and had a density of 2.13 g/cm^3^. The Na_2_SiO_3_ used was supplied by Xatico (Maizières-lès-Metz, France) and provided by the Geosil range. Geosil B47T was used with a molar ratio of Ms = SiO_2_/NaO_2_ of 1.70 and a density of 1.57 g/cm^3^. It was composed of 43.80% m Na_2_O, 37.70% m SiO_2_, and 10.20% m Al_2_O_3_.

The NaOH solution was prepared by dissolving solid NaOH pellets in the Na_2_SiO_3_ solution directly to reduce the amount of water in the mix. Depending on the mass quantity of pellets added, solutions with different concentrations could be prepared, which made it possible to modify the ratio Ms = SiO_2_/Na_2_O of the alkali reagent solution. The following formula was used to prepare our solutions:

(1) $$m_{NaOH}\;=\;\frac{n\cdot M}{\%purity}\cdot 100$$

where $m_{NaOH}$ = the mass of NaOH to be dissolved in 1 L of solution in g, with $\%Na_{2}O=m_{NaOH}/(m_{NaOH}+m_{H20}$), n = the molarity of the solution, and M = the molar mass of sodium hydroxide, which is 40 g.mol^−1^ [50].

#### 2.2.2. Supplementary Materials

Supplementary materials were used in the study to compare results obtained for a geopolymer sediment-based mortar with and without addition. The OPC used in this study is a Portland cement CEM I 52.2 N PM, supplied by Calcia (Paris, France). GGBFS was supplied by the company Ecocem (Fos-sur-Mer, France). MK was supplied by the company Argeco (Toulouse, France) and was obtained by flash calcination of kaolinitic clay. The major element compositions of each material are presented in [41].

#### 2.2.3. Mixture Design Method

Faced with the difficulty of using a general approach for the formulation of geopolymers, the formulation method chosen for the study was inspired by previous work from the authors of [54], where the SiO_2_/Na_2_O ratio of the activating solution was varied to find an optimum molarity of the NaOH solution. Other parameters were considered during formulation, such as limiting the ecological impact of the solutions by choosing a minimum alkaline reagent/precursor ratio (R/P) set at 30%. The feasibility of use on-site (safe handling of the solution) was also considered.

Thereupon, four binder formulations have been defined using untreated Pauillac sediments. The GSB, which stands for “geopolymer sediment-based binder”, is the optimal formulation found in the previous work [54], which is defined by its mass ratios of R/P = 0.31 and Na_2_SiO_3_/NaOH = 1.54 with a 4 mol.L^−1^ NaOH solution. To compare the use of alkaline reagents with conventional cement treatment for sediment stabilization, the formulation OPC was established and corresponds to the use of 10% OPC by mass of solid sediment. The binders GSB_OPC, GSB_GGBFS, and GSB_MK correspond, respectively, to binders to which 10% of OPC, GGBFS, and MK have been added. The objective is to compare the use of a cement-based hydraulic treatment to a treatment based on alkaline reagents for dredged sediments. Pauillac sediments (PAUs) were used for all the binders in the study with a water content of 30%. For each mix, the water/solid ratio was set at 0.36 as well as a constant volume of 0.98 m^3^. The water in the silicate solution and the NaOH solution was considered in the calculation as well as the initial water content of the sediment during mixing. For each formulation and curing age, tests were conducted on at least three identical specimens. Table 3 presents the mix proportions defined for each binder of the study.

The molar ratios of the geopolymerized mixtures, calculated according to the mineralogy of the sediments and the alkali reagents before the reaction, are presented in Table 4. It is important to emphasize that the reported Si/Al ratios represent the molar proportions of silicon and aluminum initially present in each mixture. However, the amounts effectively participating in geopolymerization may vary, as they depend on how extensively the different minerals within the sediment dissolve under alkaline conditions. The minimum and maximum values given in the table correspond to the intervals described in the literature [50,55,56,57]. If the SiO_2_/Na_2_O ratio was defined to correspond to the intervals, the other ratios, depending on the mineralogy of the sediments, do not satisfy the recommended values, except for the H_2_O/Na_2_O ratio. Nevertheless, they remain slightly higher or lower than these values and are in the same order of magnitude.

#### 2.2.4. Casting Specimens and Curing Conditions

The activating solution was prepared 24 h before mixing and allowed to cool at room temperature (20 ± 2 °C). The mortars were made according to the NF EN 196-1 [58] standard in a 5 L mixer. For the mixtures with binders, the sediments and binders were introduced into the mixer and mixed for 10 s. Once the materials were homogenized, NaOH and silicate solution were added at low speed for 90 s. The mixer was then stopped to remove the mortar adhering to the sides and bottom of the bowl and then resumed at high speed for 60 s. The mixtures were poured into standard 4 × 4 × 16 cm^3^ molds and then mounted with a vibrating table. The specimens were removed from the molds after 48 h and stored in a storage room at 20 °C and a relative humidity of over 60%.

### 2.3. Characterization Methods of the Binder

#### 2.3.1. Setting Times

The Vicat apparatus, as per NF EN 196-3 [59], was used to measure the setting times. The paste was introduced into the conical mold, and the penetration depth of the Vicat needle was monitored at 15 min intervals. The initial setting time corresponded to the moment when the binder first reacted with the alkaline solution, and the needle reached a penetration of 25 mm. The final setting time was defined as the point at which the needle could no longer penetrate the material.

#### 2.3.2. Semi-Adiabatic Calorimetry

Semi-adiabatic calorimetry was assessed using a Langavant calorimeter from Dye Méca (Fresnes, France) to investigate the hydration characteristics of the samples. Tests were performed following NF EN 196-9 [60]. The fresh mixtures of binders were introduced into sealed cylindrical steel containers (85 mm × 15 mm) into which a temperature probe was inserted. The temperature trend was recorded by a recording thermocouple connected to a computer data acquisition system. Measurements were taken every 10 min for 48 h.

#### 2.3.3. Compressive and Flexural Strength

Compressive strength measurements were conducted in accordance with NF EN 1015-11 [61] using an electromechanical press with a capacity of 100 kN at a constant loading rate of 0.6 mm/min. Three prismatic specimens of dimensions 4 × 4 × 16 cm^3^ were tested for each mixture after 7, 28, and 90 days.

#### 2.3.4. Porosity

The pore structure parameters were determined by using Mercury Intrusion Porosimetry (MIP). The samples were kept at 40 °C for 24 h to evaporate free water before the test. In the context of the study, the rise in pressure was carried out from 0.01 bar to 2 bar in the first porosimeter. In the second porosimeter, the pressure reached 20 bars. The results showed a porosity range from 10 nm to 150 μm.

#### 2.3.5. Scanning Electron Microscopy Coupled with Spectroscopy

Chemical characterizations were performed using SEM coupled with EDS. The observations were carried out with an SEM EVO 50 microscope on the inner part of the specimens cured for 28 days. The test was carried out by backscattering of ESB electrons on a Zeiss EVO 50 and secondary electrons on the surface with different magnifications (500× to 5000×) to observe and identify the products resulting from the interaction of precursors with alkaline reagents. Observations were made on samples of specimens cured for 28 days. For observation, the samples were first coated with a 5-nanometer layer of platinum to prevent the accumulation of static electric fields in the sample. Measures were made thanks to the Aquitaine Platform for the Characterization of Materials (PLACAMAT).

#### 2.3.6. X-Ray Diffraction

The mineralogical phases were identified using a Bragg–Brentano D8 A25 diffractometer, which had an angular range of 2θ = 5–90° and a step size of 0.02° 2θ. XRD was also used to compare the crystal structures of different formulations. The crystalline phases were identified using Profex software with an accuracy of χ^2^ < 1.5. The measurements were carried out at the Institute of Condensed Matter Chemistry of Bordeaux (ICMCB).

#### 2.3.7. Nuclear Magnetic Resonance

The spectra presented were recorded on a Bruker AVANCE III 300 MHz spectrometer (B0 = 7.05 T) (Brucker, Karlsruhe, Germany) rotating at the magic angle (MAS, rotation frequency 10 kHz) in direct polarization (single pulse, π/12, 1.1 µs) with a recycling delay of 1 s. All experiments were conducted under magic-angle spinning (MAS) conditions with identical acquisition parameters applied to all binder formulations to ensure qualitative comparability of the spectra. The samples from the 4 × 4 × 16 cm^3^ specimens were finely grounded and placed in a rotor. Measures were made thanks to the ICMCB. All NMR spectra were referenced using standard chemical shift conventions. ^29^Si chemical shifts were referenced to tetramethylsilane (TMS, 0 ppm) using an external secondary reference, ^27^Al chemical shifts were referenced to 1 M Al(NO_3_)_3_ aqueous solution (0 ppm), and ^23^Na chemical shifts were referenced to 1 M NaCl aqueous solution (0 ppm). Spectra were acquired under identical experimental conditions for all samples. For comparison purposes, NMR spectra were normalized to their maximum intensity, allowing qualitative comparison of peak positions and relative spectral evolution between binder formulations without implying quantitative phase proportions.

#### 2.3.8. Thermogravimetry Analysis

The TGA was performed using a TGA/DSC 1 STARe System by METTLER TOLEDO (Greifensee, Switzerland). Prior to analysis, all samples were oven-dried at 40 °C until constant mass was achieved to remove residual free moisture while minimizing the alteration of hydration or alkali-activation products. The dried samples were then gently ground and sieved to a particle size below 125 µm to ensure thermal homogeneity. The analysis was carried out up to 1100 °C with a heating rate of 10 °C/min under dry air with a fixed flow rate of 20 mL/min. Alumina crucibles were used, and the sample mass was approximately 90.0 ± 0.5 mg. Baseline correction was performed using an empty alumina crucible under identical experimental conditions, and all mass-loss data were normalized to the initial sample mass. The analysis was conducted using Stare software (https://www.fishersci.com/shop/products/stare-software-option-tga-evaluation/01915202, accessed on 8 August 2023) at the Canoë Platform.

## 3. Results and Discussions

### 3.1. Fresh State Properties

**Figure 2:**
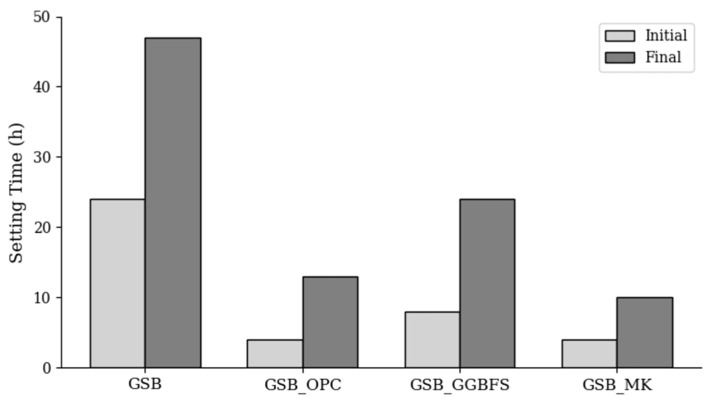
Initial and final setting of the binders studied.

One of the first indicators of binder reaction kinetics is setting time. Setting is the transition from a paste with a fluid consistency to a hardened state. It is the result of a series of mechanisms and is mainly linked to chemical reactions and the elimination of water. The beginning and end of the setting of the binders in the study are shown in Figure 2.

The GSB has an initial setting time of 24 h and a final setting time of 48 h. These long setting times, compared with cementitious materials, can lead to delayed gains in strength. They can be explained by the low reactivity of sediments to alkaline reagents, but also by the presence of organic matter and the clay phase in the sediments, which retain water and cations, thus delaying the precipitation of hydrates, the presence of which is necessary to initiate the setting process [17]. With the addition of OPC, a faster initial setting time of 4 h and a final setting time of 8 h are observed due to the hydration reactions of the cement. The setting times are similar for GSB_MK and relatively longer for GSB_GGBFS, with an end of setting at 7 h and 16 h, respectively.

**Figure 3:**
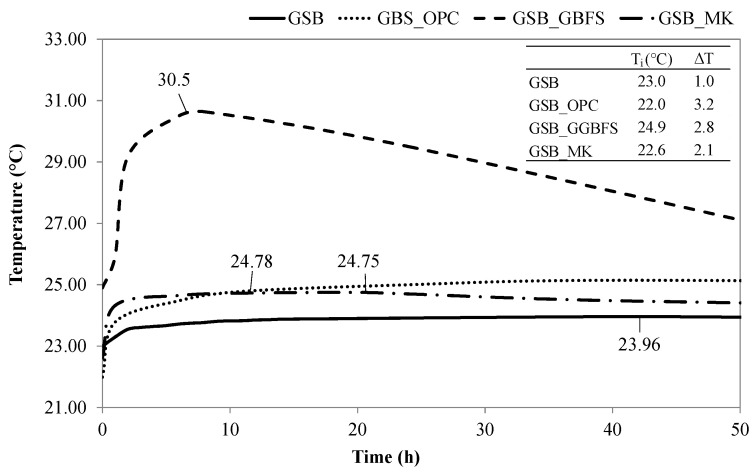
Evolution of the temperature as a function of time for the different mixtures [23].

The addition of co-products reduces setting times, possibly due to several mechanisms. First, the setting behavior is closely related to the extent of polymerization occurring within the geopolymer network [62]. Introducing MK or GGBFS modifies this process by supplying additional reactive silica and alumina, including fractions that may remain bound within the sediment matrix. As noted by Yang et al. [63], Al^3+^ ions exhibit faster dissolution in alkaline environments, contributing to reaction products that form more quickly. Likewise, a higher concentration of soluble Si^4+^ enhances the polymerization rate. Consequently, incorporating MK or GGBFS accelerates gel development and results in shorter setting times. The second phenomenon may be linked to the decrease in water. Geopolymer formation does not involve net water consumption [53], so the addition of hydraulic materials such as OPC or latent hydraulic materials such as GGBFS allows better consumption of excess water in geopolymer matrices and leads to faster setting times. Due to chemical reactions, catches are often correlated with heat release. Semi-adiabatic calorimetry data illustrate a temperature profile representative of reaction kinetics. Figure 3 illustrates the temperature evolution for each binder as well as the temperature difference between the maximum temperature and the initial temperature measured.

The reaction peaks were observed between 10 h and 42 h, which is in line with the setting times measured. The GSB reached its peak temperature later than the other formulations and exhibited a lower total heat release (ΔT), suggesting lower reactivity of sediments in the absence of supplementary materials. The improvement in uptake is therefore linked to better dissolution of the ions when the co-products are added and as the Al^3+^ and Si^4+^ contents are increased. Nevertheless, GSB_OPC and GSB_GGBFS reached maximum temperature more quickly (9 to 11 h) than GSB_MK (20 h). This difference in kinetics may be due to the presence of calcium in the matrices with OPC and GGBFS. The release of Ca^2+^ ions, which is faster than that of Al^3+^ and Si^4+^ ions in an alkaline medium, could lead to a faster setting time. The faster kinetics observed here for GSB_OPC and GSB_GGBFS can be explained by the hydration phenomenon of the co-products, but also by the release of Ca^2+^ ions, which may have led to the formation of a C-S-H gel in combination with a geopolymer gel. The geopolymerization reactions for the GSB and GSB_MK are much delayed due to the presence of water in the matrices and the low presence of Ca^2+^ ions.

### 3.2. Mechanical Properties

**Figure 4:**
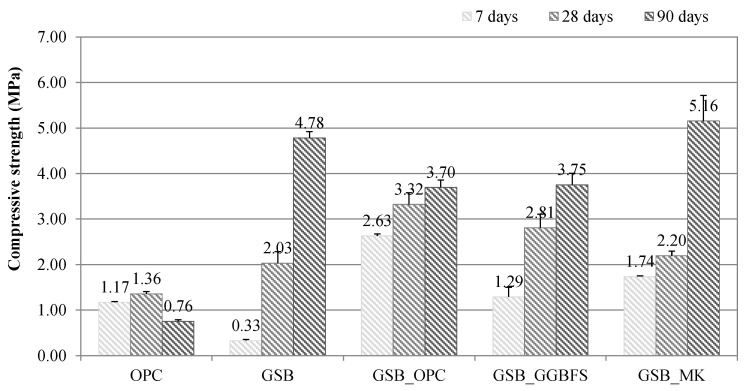
Compressive strength values of specimens [23].

The reaction kinetics at a young age were used to predict the mechanical behavior of the formulations. To justify these conclusions and validate the mechanical performance of the formulations, compression and flexural tests were carried out at different times. Figure 4 shows the strength values obtained at 7, 28, and 90 days.

The GSB formulation achieves a low strength at 7 days (0.3 MPa), which is consistent with the delayed setting times and low early age heat release observed by calorimetry. This behavior reflects the limited initial dissolution of aluminosilicate phases from untreated sediments under low-alkali conditions. The OPC formulation, i.e., the mixture of sediments treated with cement, has a compressive strength three times higher at 7 days (1.2 MPa), driven primarily by conventional cement hydration reactions rather than sediment participation.

With the addition of co-products, an increase in compressive strength was observed at a young age. At 7 days, compressive strengths increased by 87% with the addition of OPC, 74% with the addition of GGBFS, and 81% with MK compared with the GSB. These early age gains are attributed to enhanced ionic availability (Ca^2+^, Si^4+^, and Al^3+^), which accelerates the precipitation of binding phases. In particular, the rapid strength development of GSB_OPC is linked to the formation of calcium silicate hydrate (C-S-H), which dominates the early binding mechanism. However, for GSB_GGBFS, despite a relatively high calcium content and higher heat release observed by calorimetry, the compressive strength at 7 days remains limited (1.3 MPa). This suggests that early thermal activity does not directly translate into effective load-bearing gel formation, highlighting the complexity of reaction pathways in sediment-rich systems.

While the OPC formulation exhibited only marginal strength gains at 28 days, the geopolymerized specimens demonstrated significant improvements compared to their 7-day values. The highest increase was observed for the GSB specimen, whose value rose by 84% to 2 MPa. Linked to the mechanisms observed at a young age, specimens with high Si^4+^ and Al^3+^ contents and a low Ca^2+^ content have delayed reactivity. Geopolymer gel formation is established over a longer period compared with systems with high Ca^2+^ content. The mechanical strengths of specimens with co-products are also significantly higher at 28 days: GSB_OPC reaches 3.3 MPa, GSB_GGBFS reaches 2.8 Mpa, and GSB_MK reaches strength of 2.2 MPa, which is similar to the GSB.

**Figure 5:**
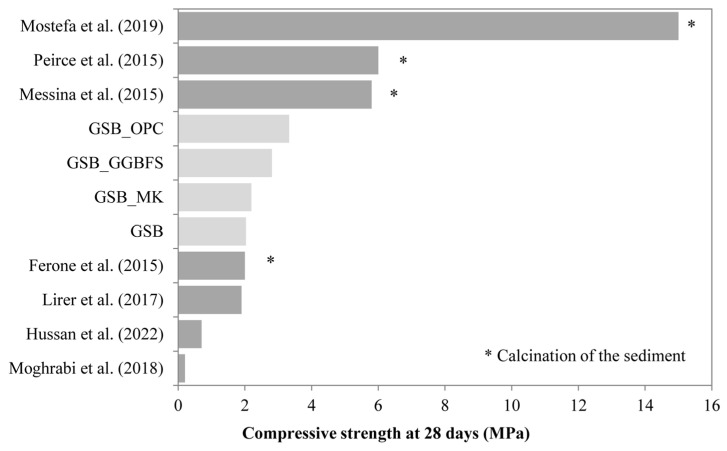
Histogram of compressive strengths reported in studies valuing sediments in geopolymer matrices [18,21,22,23,26,30,31,33].

To situate the values obtained from the literature, Figure 5 presents the compressive strength values extracted from studies on the geopolymerization of sediments. The studies were selected if the percentage by mass of sediment in the matrices was greater than 70%.

The mechanical strengths obtained for our formulations are similar to the strengths obtained with calcined sediments, except for the study by Mostefa et al. [21], where the higher mechanical strength obtained is obviously linked to the calcination of the sediments, which makes them more reactive, but also to the use of a high concentration NaOH solution (8 mol.L^−1^ to 12 mol.L^−1^) combined with a thermal cure at 60 °C. The values of 6 MPa obtained by the study of Peirce et al. [22] and Messina et al. [31] are close to the values obtained for our formulations at 90 days. The relevance of searching for an optimal formulation using several tools is illustrated here.

**Figure 6:**
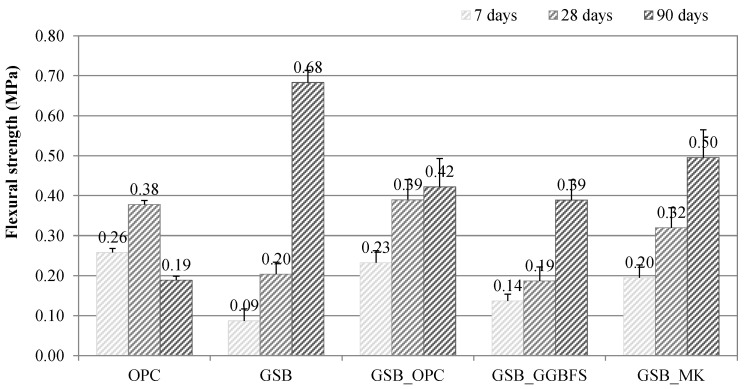
Flexural strength values of specimens [23].

The results at 90 days show different trends to those at previous ages. Firstly, for OPC, cement treatment leads to compressive strength failure at 90 days. This may be related to the presence of organic matter in the sediments, which has been reported in numerous studies as a detrimental factor in cement hydration processes [7,64,65]. While the clay present in the sediments seems to be a hindrance to the development of strength for OPC, it is, on the contrary, an advantage for specimens based on alkaline reagents, particularly for GSB and GSB_MK, which achieve strengths of 4.8 MPa and 5.2 MPa, respectively. The GSB_OPC and GSB_GGBFS samples both achieve a similar value of 3.7 MPa at 90 days. The addition of a co-product with high calcium content limits strength gains beyond 90 days. Several studies have shown that incorporating calcium-rich materials into geopolymer systems may hinder long-term strength development [66,67]. This is because geopolymerization relies primarily on the dissolution and subsequent reaction of Si^4+^ and Al^3+^ ions, which govern the formation of the aluminosilicate network. Thus, if the amount of calcium is insufficient to lead to the formation of a stable C-S-H gel, coexistence with the aluminosilicate geopolymer gel will lead to poor long-term mechanical properties [66]. Calcium, therefore, plays little part in the final nature of the binder, as reported by Zhao et al. [67], despite higher strength gains at a young age. The bending strength values are shown in Figure 6. The conclusions drawn above apply to the bending results. However, at 90 days, the GSB has a higher flexural strength than GSB_MK.

The compressive strength results show that sediments treated with alkaline reagents perform better than those treated with cement. The mechanical strength of OPC degrades over time, whereas the alkaline reagent-based specimens increase their strength considerably at 90 days. The superior long-term performance of calcium-poor systems indicates that sustained geopolymerization, rather than early hydration, governs mechanical durability in sediment-rich binders. Furthermore, for our systems, the addition of high-calcium co-products has a detrimental effect beyond 28 days. These results are in line with part of the literature, although most articles support the beneficial effect of calcium in geopolymer matrices [68,69,70,71]. This discrepancy is attributed to the combined effects of low alkali molarity, clay-rich sediment matrices, and incomplete calcium incorporation into a stable binding network. Calcium, therefore, plays a dual and context-dependent role: it accelerates early age reactions but, under the present formulation conditions, disrupts long-term geopolymer network development, leading to inferior mechanical performance at extended curing times.

**Figure 7:**
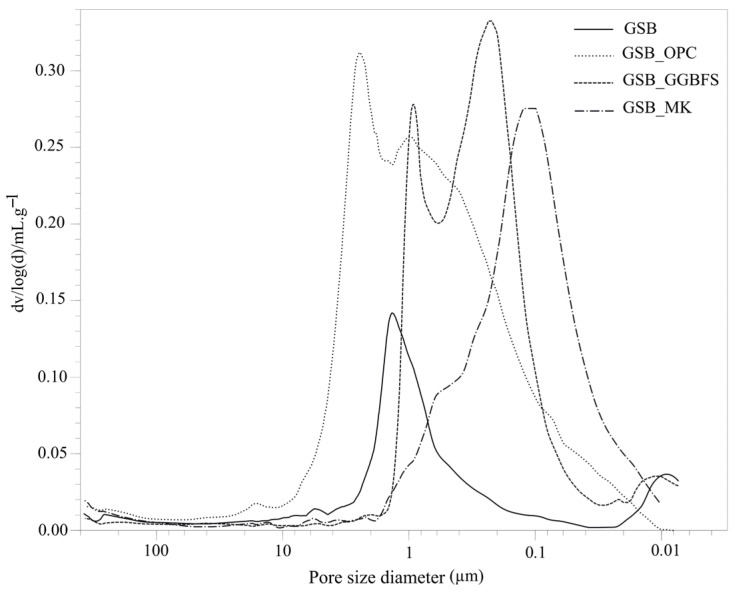
Pore size distribution of the cementitious binder measured using mercury intrusion porosimetry [23].

**Figure 8:**
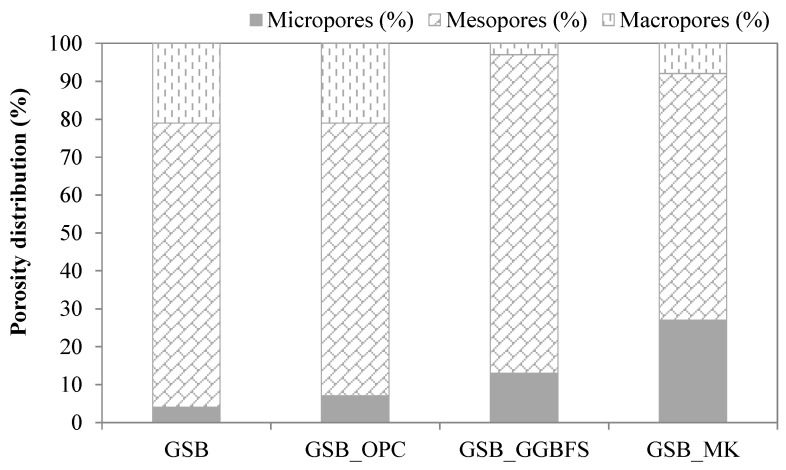
Distribution of the porosity of the formulations at 28 days [23].

The pore structures of the binders, measured at 28 days and shown in Figure 7, provide further insight into the observed mechanical behavior. Porosity is a key microstructural parameter controlling strength evolution and durability in geopolymer systems, particularly in heterogeneous matrices derived from natural sediments. The pore size profiles of the four mixtures extended from 10 µm down to 0.01 µm. Incorporating OPC or GGBFS broadened this distribution, producing ranges of approximately 7–4 µm and 4–0.01 µm when compared with the GSB reference. More specifically, GSB_OPC exhibited a dominant pore interval between 6 and 0.9 µm, whereas GSB_GGBFS showed a finer distribution spanning from 0.9 to 0.01 µm. In contrast, adding MK refined the pore network, resulting in a more uniform structure and shifting the macropores present in the GSB toward smaller pore sizes. These results may imply a different nature of gel in the structure depending on the co-product used [67]. To better characterize the evolution of porosity as a function of the co-product used, Figure 8 presents the pore distribution categorized into three size ranges, defined by Anderson and Pratt [72], macropores larger than 50 nm, mesopores between 50 and 2 nm, and micropores smaller than 2 nm.

Table 5 shows the total open porosity of the materials and the proportion of the different classes. Incorporation of OPC or GGBFS increases total porosity relative to the GSB, despite higher early age strength. This apparent contradiction highlights that early mechanical performance is not governed solely by porosity magnitude, but by pore connectivity and gel continuity. GSB_MK exhibits a refined pore structure with a higher proportion of microporosity, indicative of a more homogeneous and interconnected geopolymer gel network, which explains its superior long-term strength.

The addition of OPC does not greatly modify the porosity distribution of the GSB. On the other hand, GSB_OPC has a higher porosity of 51% compared to GSB, which was at 23% without the addition of the co-product. This could be related to the faster setting time for GSB_OPC, which led to rapid water consumption, inducing the formation of microcavities, which can reduce long-term mechanical strengths, as previously observed. GSB_GGBFS and GSB_MK also have higher porosities than GSB, but there is a greater distribution of microporosity and less macroporosity.

Observation of the differences in porosity distribution according to the co-products used confirms the differences in reactions between the different formulations. In fact, studies have shown that the simultaneous formation of geopolymer gel and hydrated calcium gel is possible in a single binder [73,74]. In view of the porosities and mechanical strengths observed, the existence of the following two families of binders for our formulations is noted: binders in which a geopolymer gel, formed by the reaction of sediments with alkalis, and a gel formed by the hydration of calcium coexist (GSB_OPC and GSB_GGBFS) and binders in which only the geopolymer gel predominates (GSB and GSB_MK). The refinement of the porosity of GSB with the addition of MK shows that there was a synergy between the MK and the sediments towards the formation of a geopolymer gel (N-A-S-H). The porosity values seem to indicate that the resistance of GSB_OPC is linked to the coexistence of the two gels. Indeed, when the NaOH concentration is low, the dissolved calcium from OPC can form an amorphous C-S-H gel without participating in the geopolymerization reactions. The same conclusion could be applied to GSB_GGBFS, but its porosity is closer to GSB_MK than to GSB_OPC. Alonso and Palomo [75] reported that, in certain alkaline environments, the presence of Ca^2+^ ions could lead to the formation of a N-(C)-A-S-H aluminosilicate gel whose characteristics are the same as a geopolymer gel. The lower presence of calcium in GGBFS compared with the cement used is responsible for this phenomenon. If the calcium content is too low, the reaction of Si^4+^ and Al^3+^ ions may inhibit the formation of C-S-H gel. The free calcium in GSB_GGBFS, therefore, did not form a C-S-H gel, but was able to actively participate in the formation of a N-(C)-A-S-H gel.

### 3.3. Microscopic Properties

**Figure 9:**
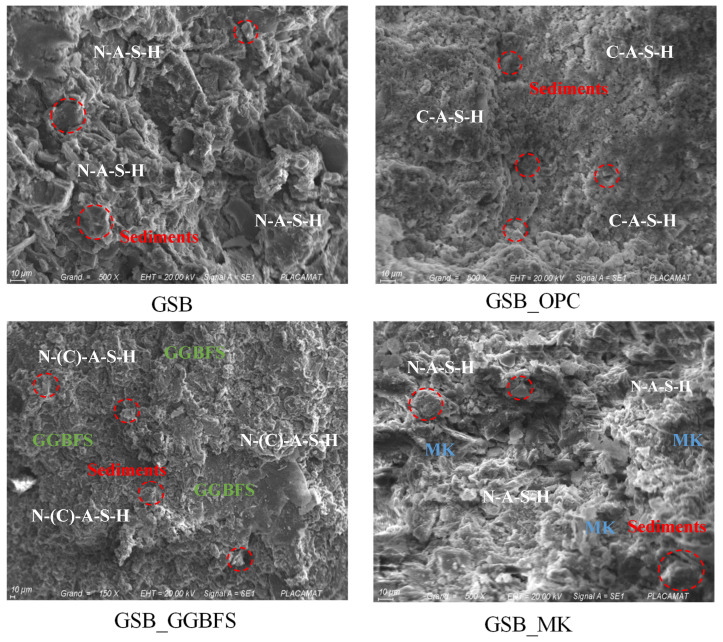
SEM observations at 10 µm from the specimens [23].

SEM and EDS analyses were carried out to link macroscopic observations to the microstructural properties of the specimens. Morphometric observations revealed differences between the structures. Figure 9 shows the SEM images obtained at low magnification (×500) for the four formulations.

At equal magnification, topological differences between the matrices are observed. For GSB and GSB_MK the SEM, observations show the formation of a dense and gelled matrix around the particles of MK or sediments. These matrices are like what other studies have observed [18,19,76,77] and testify to the formation of a N-A-S-H geopolymer gel. Morphological differences are, however, noticeable for the two specimens. GSB exhibits a less developed topology than GSB_MK, which exhibits smooth surfaces, indicating better geopolymer formation. For GSB_OPC, the matrix is different and is less gelled. A topology like that of cement is observed with the formation of a C-S-H product [78,79]. The gel forms around the sediment grains, trapping them in the C-S-H matrix. The formation of C-S-H limits the reaction of sediments during the introduction of alkaline reagents. For GSB_GGBFS, the topology differs from the preceding formulations, and one observes a mixed topology which resembles that of GSB_OPC and that of GSB_MK. In general, it is observed that the precursors retain their initial shape during the geopolymerization process, thus showing that the geopolymeric reaction takes place mainly at the level of the surface layer of the solid particles.

**Figure 10:**
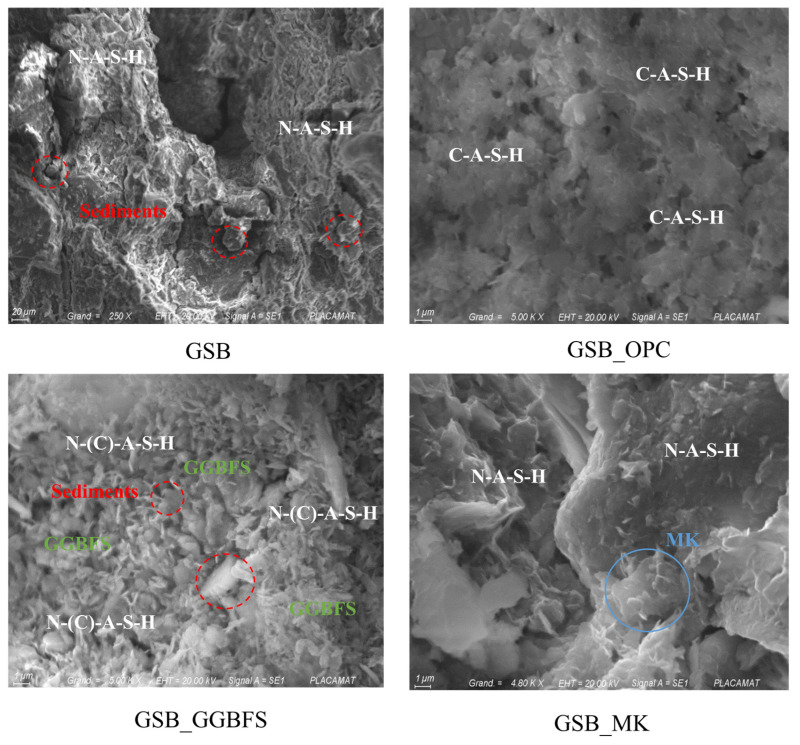
SEM observations at 1 µm from the specimens [23].

To observe more precisely the matrix products developed on the solid particles, Figure 10 shows the SEM images obtained at a higher magnification. For GSB_OPC, the matrix observed shows some porosity, but the matrix is dense and homogeneous. For GSB nomenclature specimens, a gel formation is observed on the solid particles of the grains. Gel development for GSB_GGBFS seems to occur around the GGBFS grains.

**Figure 11:**
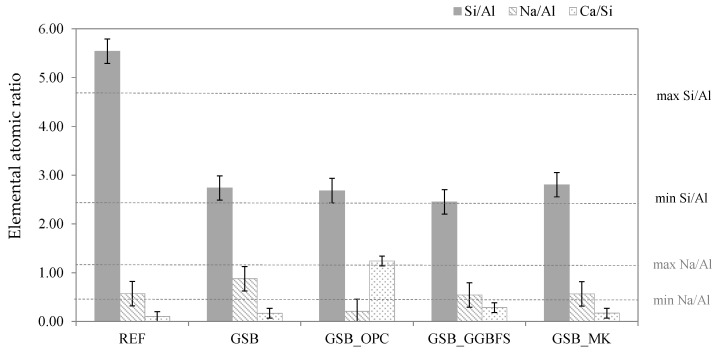
Relative evolution of binder ratios compared to the initial GSB mix at 28 days [23].

To determine the elemental composition of the products observed, the evolution of the elemental ratios obtained by EDS is presented in Figure 11. The maximum and minimum values of the ratios, as defined in the literature, are also shown. A clear decrease in the Si/Al ratio is observed for all formulations. This decrease indicates that the Al^3+^ and Si^4+^ ions naturally present in the sediments and co-products were consumed in favor of geopolymerization [23]. It should be noted that, despite the absence of co-products, the Si/Al ratio for GSB, which contains only sediments as precursors, also decreases, indicating that the sediments are not inert and are actively involved in geopolymerization. The Si/Al ratios after the reaction correspond to the ratio indicated in the literature and thus confirm the defined ratios. Moreover, this ratio also decreases for GSB_OPC, which could indicate that the development of a C-S-H gel did not completely interfere with the dissolution of Si^4+^ and Al^3+^ ions from the sediments, which formed a secondary aluminosilicate gel with high C-A-S-H calcium content. The Na/Al ratio remained fixed for GSB_GGBFS and GSB_MK, since the addition of co-products increased the quantity of Al ions. On the other hand, the Na/Al ratio changes for GSB due to the addition of Na_2_SiO_3_, which increases the quantities of Na in the solution. For GSB, the Na/Al value is closest to one, which is the optimum value for this ratio. This explains the better compressive strengths than GSB_OPC and GSB_GGBFS at 90 days. For GSB_OPC, the ratio decreases, indicating that Al^3+^ must have been consumed in favor of the formation of a C-A-S-H gel.

Finally, the Ca/Si ratio was also studied, despite its lack of optimum in the literature. For GSB and GSB_MK, this ratio remained unchanged in relation to the reference, confirming the unique formation of an aluminosilicate geopolymer gel. On the other hand, there was a slight increase for GSB_GGBFS due to the addition of GGBFS and the formation of a N-(C)-A-S-H gel. A major increase is observed for GSB_OPC due to the addition of cement, which disrupted the formation of sediment geopolymer by transforming the geopolymer gel into a C-A-S-H gel.

**Figure 12:**
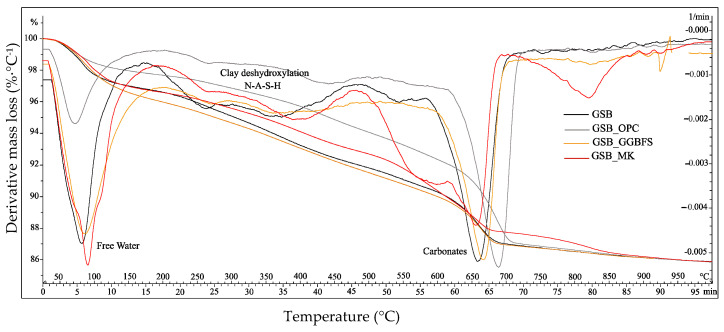
TGA of the studied binders [23].

**Figure 13:**
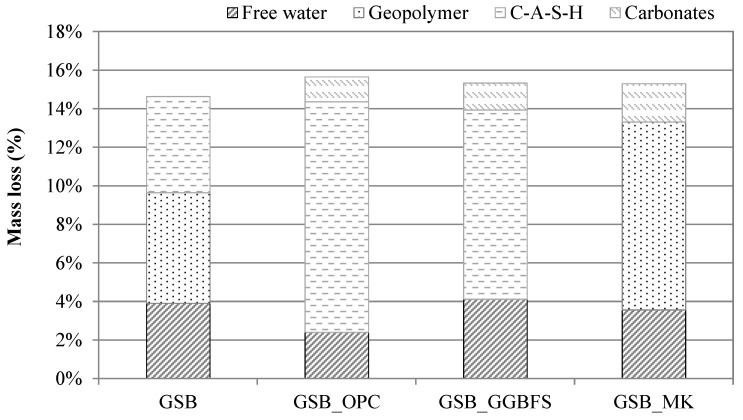
Histogram of mass losses linked to different phases for each binder [23].

TGA was employed as a comparative tool to evaluate the relative mass-loss behavior associated with gel formation and phase evolution within the different binder systems [23]. Given the known overlap of thermal events in alkali-activated materials, interpretation of mass-loss regions is considered semi-quantitative and intended to support comparative discussion rather than absolute phase quantification. Phase assignments are therefore discussed in conjunction with SEM-EDS, XRD, and NMR results rather than as standalone evidence. TGA was carried out at 28 days for the binders and is shown in Figure 12. To facilitate interpretation and ensure reproducible quantification, the TGA curves were divided into distinct temperature regions based on commonly reported thermal responses of alkali-activated and sediment-based binders. A low-temperature region from 50 to 200 °C was associated with the loss of free and weakly bound water, an intermediate-temperature region from 200 to 600 °C with dehydroxylation and progressive decomposition of aluminosilicate geopolymer gels, and a high-temperature region above 600 °C with carbonate decomposition. For quantitative comparison, the mass-loss values reported in Table 6 and illustrated in Figure 13 were calculated by numerical integration of the TGA curves over fixed temperature intervals, namely, 50–100 °C for free and weakly bound water, 100–200 °C for the dehydration of calcium-bearing hydrates (C-A-S-H or N-(C)-A-S-H), 200–600 °C for the dehydroxylation and progressive decomposition of aluminosilicate geopolymer gels (N-A-S-H), and 600–800 °C for carbonate decomposition. Mass losses were determined as the relative weight difference between the onset and end temperatures of each interval, ensuring direct comparability between the different binder systems [23].

At the low-temperature region, the first major losses of mass, characterized by sharp peaks observed between 70 °C and 90 °C, correspond to the evaporation of free water at the surface and physically adsorbed or weakly bound water trapped within the pore network of the geopolymer gel and sediment particles. The associated mass losses are of the order of 2.4% (GSB_OPC), 3.2% (GSB), 3.6 (GSB_MK), and 4.1% (GSB_GGBFS). In view of the low water losses for GSB_OPC, a denser structure was formed, which led to better resistance at a young age. However, this reduced mass loss is attributed to the rapid precipitation of calcium-rich hydrates rather than to the formation of a continuous aluminosilicate geopolymer network. Nevertheless, the mass losses at this temperature for the other specimens are similar to those in the literature and indicate good polycondensation of the gels formed. The higher water loss for GSB_GGBFS may reflect a less stable gel structure and a more open pore network, consistent with porosity measurements. Between 100 °C and 200 °C, a slight shoulder in mass loss was observed for GSB_OPC and GSB_GGBFS, linked to the introduction of calcium into the geopolymer matrices. They may be associated with the formation of C-A-S-H or N-(C)-A-S-H [76,80]. This feature is absent or weak in calcium-poor systems, confirming different reaction pathways depending on calcium availability. The strength gains for these formulations are therefore linked to the formation of C-A-S-H or N-(C)-A-S-H gel.

In the intermediate temperature region, the weight loss was attributed to progressive dehydroxylation and structural rearrangement of aluminosilicate binding gels, together with contributions from residual clay minerals and organic matter. The dehydroxylation of the clay phases of the sediment, particularly the disordered kaolinite, occurs between 530 °C and 570 °C [81,82], accompanied by condensation of the hydroxyl group and degradation of the organic matter. Corresponding mass losses are generally due to the release of water by condensation/polymerization of Si-OH and Al-OH groups as an aluminosilicate network formed from the reaction of two hydroxyl groups [83]. Thus, there is a progressive decomposition of the geopolymer gel structure formed during curing of the order of 5.7% for the GSB and 9.5% for GSB_MK, suggesting a higher degree of aluminosilicate gel development for GSB_MK. In some studies, this mass loss is used as a relative measure of the degree of reaction in geopolymerized systems, which confirms the resistance obtained for GSB_MK, suggesting a higher degree of aluminosilicate gel development for GSB_MK.

In the high temperature region, the final major peak around 650 °C to 750 °C is attributed to bond failure and collapse of the clay structure of the sediments and decarbonation of the calcite [84]. For GSB_OPC, GSB_GGBFS, and GSB_MK, partial involvement of carbonate phases in gel formation may influence the position and intensity of this peak. At temperatures above ~900 °C, recrystallization of geopolymer gels or thermally modified clay phases into more stable crystalline phases may occur, generally without additional mass loss.

The distinct thermal signatures observed among the binders confirm that calcium-rich systems are dominated by early age hydrate formation, whereas calcium-poor systems promote progressive aluminosilicate gel development that governs long-term mechanical performance.

**Figure 14:**
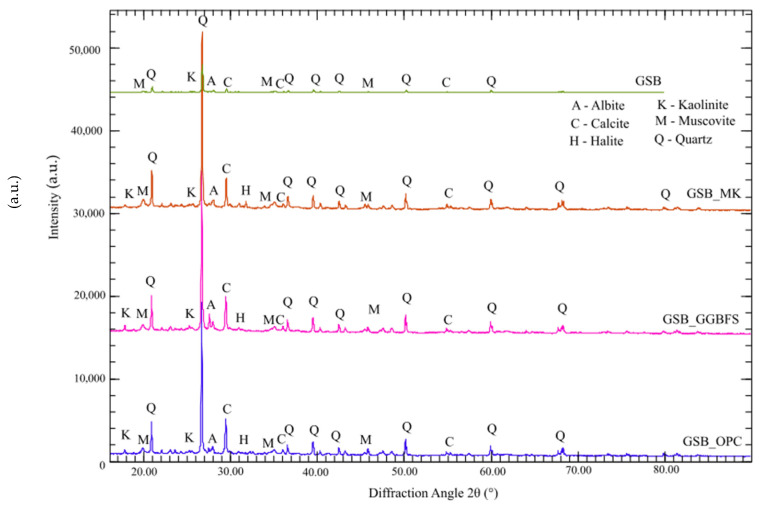
X-ray diffraction patterns of the studied binders after 28 days of curing. Identified crystalline phases are indicated as follows: Q = quartz (SiO_2_), C = calcite (CaCO_3_), K = kaolinite (Al_2_Si_2_O_5_(OH)_4_), M = muscovite (KAl_2_(AlSi_3_O_10_)(OH)_2_), A = albite (NaAlSi_3_O_8_), and H = halite (NaCl), [23].

XRD analyses were carried out to identify mineralogical differences between the binders and to observe the appearance of new phases. XRD analyses were performed on powdered binder samples using a PANalytical X’Pert Pro powder diffractometer (Malvern Panalytical, Malvern, United Kingdom) with Cu Kα radiation. Prior to analysis, hardened samples were gently crushed, finely ground, and sieved to <125 µm to ensure representative sampling and minimize preferred orientation. Phase identification was carried out using the Profex software [23]. The same acquisition parameters were applied to all binder formulations to ensure direct comparability of the diffractograms. The diffractograms of the binders at 28 days are shown in Figure 14.

The limited visual differences observed between the XRD diffractograms indicate that the primary crystalline phases of the sediments remain largely unchanged following alkali activation. This behavior is typical of alkali-activated systems, in which the main binding phases are predominantly amorphous and therefore not directly detectable by conventional XRD. Consequently, XRD is used here as a qualitative tool to monitor changes in crystalline phases, while mechanistic interpretation of gel formation and phase evolution relies on combined analysis with SEM–EDS and NMR results rather than on XRD alone. A comparison between the GSB diffractogram and those obtained after the addition of co-products reveals only minor variations. The initial mineralogical assemblage of the Pauillac sediments remains largely preserved despite alkali activation, as the intensities of crystalline phases, such as albite, calcite, halite, muscovite, and quartz, do not show significant changes. Similar observations were also made by Yip et al. [66] and Zhang et al. [85].

The absence of kaolinite peaks in the GSB system suggests partial alkali attack of clay minerals and their involvement in geopolymerization [53,86]. In contrast, the persistence of kaolinite reflections in binders containing co-products indicates that gel formation preferentially involves added aluminosilicate or calcium-bearing phases rather than extensive dissolution of sedimentary clays. This behavior is consistent with a surface-controlled alkali activation mechanism involving partial clay dissolution, rather than classical metakaolin-based geopolymerization.

The lack of newly formed crystalline phases confirms that alkali activation does not induce direct recrystallization of sediment minerals. Instead, mechanical performance arises primarily from the formation of amorphous binding gels, which are not directly observable by XRD due to their low crystallinity and the strong diffraction signal of quartz.

A broad diffuse halo observed between 22° and 26° 2θ for the alkali-activated binders reflects the presence of poorly crystalline or amorphous reaction products formed during activation. Such a feature is commonly reported in alkali-activated materials and geopolymer systems; however, it does not allow unambiguous identification of specific binding phases, such as C-S-H, C-A-S-H, or N-A-S-H, by XRD alone. Consequently, the halo is interpreted qualitatively as evidence of amorphous gel formation, while the nature and chemistry of the binding phases are inferred from complementary SEM–EDS and NMR analyses.

The use of NMR is relevant to the study of the structure of amorphous products resulting from reactions between precursors and alkaline reagents. NMR spectroscopy was used to qualitatively assess local coordination environments of Si, Al, and Na within the reaction products [23]. In the absence of peak deconvolution and quantitative fitting, the spectra are interpreted in terms of coordination trends and relative chemical shift evolution, rather than absolute phase proportions or definitive phase identification.

**Figure 15:**
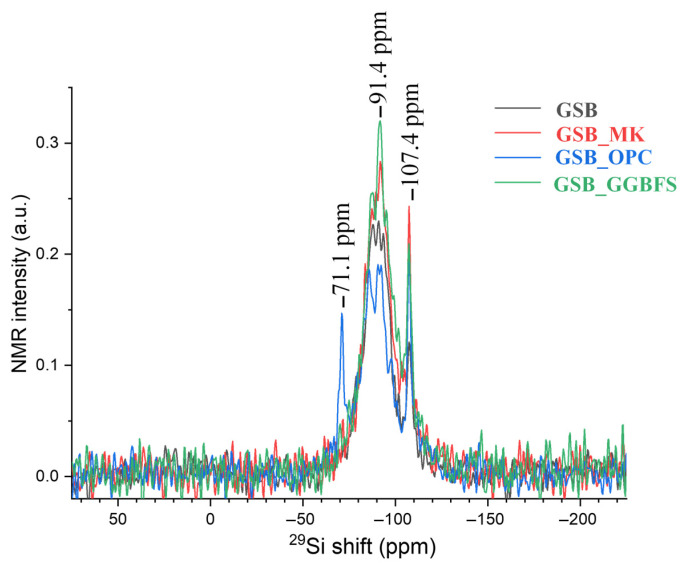
^29^Si NMR of each binder acquired under MAS conditions [23].

Davidovits was the first to study the structure of metakaolin-based geopolymers using NMR in the 1980s [14]. The ^29^Si NMR spectra presented in Figure 15 shows several intensity peaks, including a broad band between −82 ppm and −92 ppm with a maximum of −91.4 ppm. In the case of NMR analyses of geopolymers and alkali-activated systems, this broad intensity band is commonly associated with tetrahedrally coordinated silicon environments [87,88,89].

**Figure 16:**
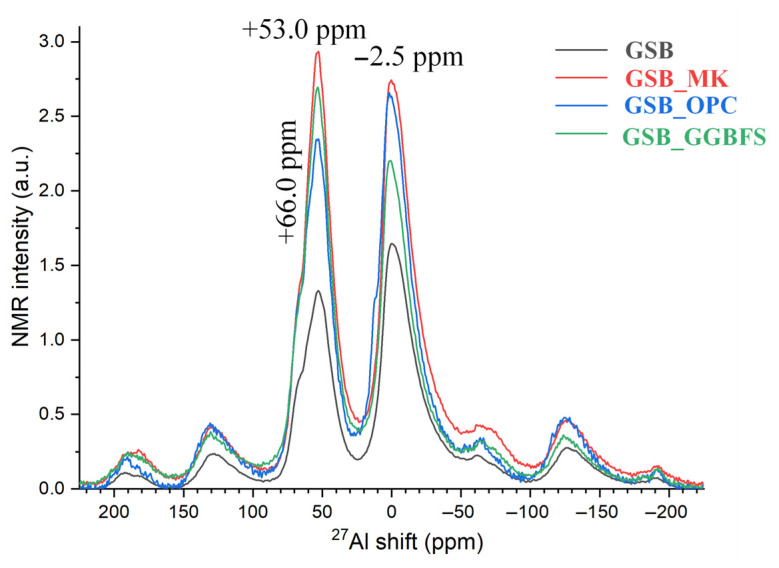
^27^Al NMR of each binder acquired under MAS conditions [23].

These contributions are generally attributed to the environments Q^2^(1Al), Q^3^(0Al), Q^4^(4Al), Q^4^(3Al), Q^4^(2Al), and Q (0Al). The observed chemical shift evolution may be influenced by a partial alkali attack of kaolinite and redistribution of Al^3+^ ions within the aluminosilicate gel networks. Through alkali activation, chemical shifts associated with clay-derived environments tend to evolve toward more polymerized Q^4^-type environments, which is consistent with the formation of geopolymer-type binding phases. However, the exact nature and proportion of the gels formed cannot be unambiguously determined from ^29^Si NMR alone, as several sites may overlap in Q^4^ type environments. The simultaneous presence of different binding environments, including C-A-S-H-type and N-A-S-H-type structures, has been reported in the literature for similar systems [87,88]. Calcium addition to aluminosilicate systems has been shown to modify N-A-S-H gels toward N-(C)-A-S-H-type environments via ion-exchange mechanisms, in which Ca^2+^ partially replaces Na^+^ [89]. These mechanisms provide a qualitative framework for interpreting the observed spectral trends, but do not allow definitive phase discrimination in the absence of spectral deconvolution. The spectra for the GSB and GSB_MK show the formation of N-A-S-H, while GSB_GGBFS shows similar displacements. This displacement may be associated with the presence of N-(C)-A-S-H. Only observable for GSB_OPC, the peak at −71.1 ppm corresponds to the formation of a C-S-H gel, showing that the following two gels coexist in the matrix: C-S-H and N-A-S-H. Buchwald et al. [90] reported the simultaneous presence of N-A-S-H and C-S-H geopolymers in their material. The ^29^Si NMR spectra also shows a peak at −107.4 ppm assigned to the Q^4^ (0Al) coordination, which corresponds to the quartz present in the sediments and is confirmed by XRD [17,22]. Figure 16 shows the ^27^Al NMR spectra of the binders.

Two bands are present with a maximum of around 53 ppm and −2.5 ppm, respectively. The first band corresponds to a shift between 70 ppm and 50 ppm. This broad signal is attributed to the tetrahedral Al^IV^, which is incorporated in N-A-S-H and N-(C)-A-S-H gels [87,91,92]. It is also associated with feldspars present in the sediments, such as albite and muscovite. Bernal et al. [93] observed, in ^27^Al NMR, a strong contribution to a high chemical shift in the C-A-S-H gel and another to a lower chemical shift in the N-A-S-H gel. Both phases are thought to contain Al in non-complete tetrahedral environments, leading to the broad resonance centered around 58 ppm or, in our case, 53 ppm. Similarly, the study by Puertas et al. [71] shows a broad resonance zone centered at 65 ppm that is attributed to Al in significantly distorted tetrahedral environments within poorly crystallized C-A-S-H and N-A-S-H. In 27Al NMR, the peaks located between 55 and 68 ppm are characteristic of the raw slag and could indicate that it has not been fully reacted. MK is also present at around 60 ppm (Al^IV^) and 0 ppm (Al^VI^), which corresponds to the bands observed here. The second band between 0 and 4 ppm (Al^VI^) suggests the possible presence of kaolinite/metakaolin that has not been dehydroxylated and therefore partially reacts during alkaline attack.

**Figure 17:**
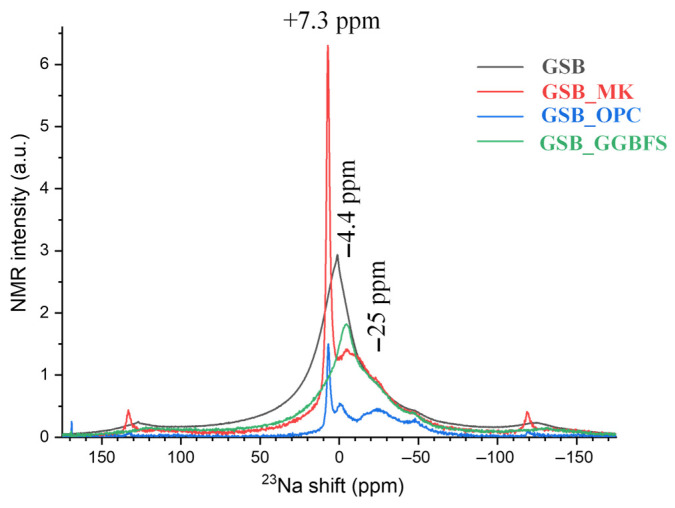
^23^Na NMR of each binder acquired under MAS conditions [23].

The ^23^Na NMR spectra, shown in Figure 17, shows a major resonance centered at around −5 ppm, which is attributed to the Na cations associated with the centered aluminum tetrahedra, playing a charge-balancing role within the frameworks of the various gels formed.

**Figure 18:**
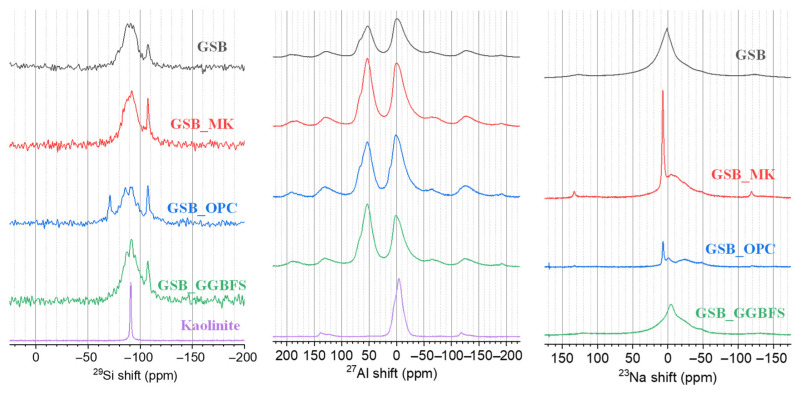
NMR spectra of binders based on different aluminosilicate sources acquired under MAS conditions [23].

Figure 18 summarizes the different spectrographs obtained. Based on the previous observations, the NMR spectra confirmed the coexistence of three types of gel in the binders studied. These results agree with those of Garcia-Lodeiro et al. [73], who also observed N-A-S-H and N-(C)-A-S-H in their matrix. The results also showed that alkaline etching was not sufficient to dissolve all the precursor ions and may have led to incomplete gel formation.

By combining mechanical testing with thermal, mineralogical, and spectroscopic analyses, clear structure–property relationships emerge for the studied binders. Calcium-rich systems (GSB_OPC and GSB_GGBFS) exhibit pronounced low-temperature mass losses in TGA and distinct shoulders between 100 and 200 °C, consistent with early formation of calcium-bearing hydrates. These systems also display limited amorphous phase evolution in XRD and NMR, reflecting rapid but heterogeneous gel formation, which explains their higher early age strength but limited long-term mechanical performance. In contrast, calcium-poor systems (GSB and GSB_MK) are characterized by broader mass-loss regions between 200 and 600 °C in TGA, indicative of progressive aluminosilicate gel development, combined with predominantly amorphous XRD patterns and NMR signatures consistent with more polymerized aluminosilicate environments. This microstructural evolution correlates with slower early age strength gain but continuous mechanical improvement at later curing ages. These observations demonstrate that long-term mechanical performance is governed not by early reaction kinetics alone, but by the extent, continuity, and chemistry of the amorphous binding gel network formed during alkali activation.

## 4. Conclusions and Perspectives

This study assessed the feasibility of using untreated dredged sediments from Pauillac as active precursors for geopolymer binder development, based on a comprehensive physical, chemical, mechanical, and microstructural characterization. The primary objective was to demonstrate that such sediments, without any thermal or chemical pre-treatment, can actively participate in geopolymerization rather than behaving as inert fillers. Additionally, the effect of incorporating various co-products on reaction kinetics, gel chemistry, and mechanical performance was evaluated.

Macroscopic performance confirmed that Pauillac sediments are not inert in alkaline environments. Their aluminosilicate composition and clay fraction support progressive dissolution and geopolymer gel formation, enabling mechanical strengths comparable to those reported for systems based on calcined sediments, despite the use of low alkali concentration and ambient curing conditions.

Alkali activation of sediments resulted in binders with superior long-term mechanical performance compared to cement-based stabilization. While cement-treated specimens exhibited rapid early age strength gain, their mechanical performance deteriorated at extended curing times, likely due to interactions between hydration products, organic matter, and clay minerals. In contrast, alkali-activated systems showed continuous strength development up to 90 days, demonstrating enhanced compatibility with sediment constituents.

Microstructural and spectroscopic analyses (SEM–EDS, TGA, XRD, and NMR) revealed that the kaolinite present in the sediments plays a key role in geopolymerization, contributing to the formation of aluminosilicate binding phases. Calcium availability was shown to strongly influence gel chemistry and reaction pathways. Calcium-poor systems favored the development of homogeneous N-A-S-H gels, whereas partial calcium addition (GGBFS) promoted N-(C)-A-S-H gel formation. High calcium content (OPC) led to the coexistence of C-S-H and N-A-S-H gels, resulting in microstructural heterogeneity and limited long-term strength development.

Porosity analysis confirmed that long-term mechanical performance is governed not only by total porosity, but by pore refinement and gel continuity. The refined microporous structure observed for the sediment–metakaolin system explains its superior strength evolution, while calcium-rich systems exhibited higher total porosity and heterogeneous pore networks.

Overall, this work provides direct experimental evidence that untreated dredged sediments can serve as reactive geopolymer precursors, offering a low-impact and resource-efficient pathway for sediment valorization. The developed binders exhibit mechanical properties suitable for public works applications, particularly for on-site implementation near port infrastructures, where transport and processing constraints are critical.

Nevertheless, the coexistence of multiple gel phases, incomplete precursor dissolution under low-alkali conditions, and the variability of natural sediments highlight the need for further investigation. Future work will focus on optimizing formulation parameters, evaluating sediment variability across the Gironde estuary, conducting comprehensive durability and environmental performance assessments, and performing life-cycle analyses to quantify the full sustainability potential of sediment-based geopolymer binders.

## Figures and Tables

**Table 1:** Physical properties of Pauillac sediments.

Properties	Value
Initial water content (%)	145.02
Blaine fineness (m^2^/g)	0.65
Specific gravity (g/cm^3^)	1.45
Plastic Limit (%)	16.60
Liquid Limit (%)	42.35
VBS	2.80
Organic content (%)	1.05

**Table 2:** Oxides composition of Pauillac sediments.

Oxides	Composition (wt. %)
Silicon Dioxide (SiO_2_)	41.38
Aluminum Oxide (Al_2_O_3_)	14.19
Iron Oxide (Fe_2_O_3_)	8.57
Calcium Oxide (CaO)	4.58
Potassium Oxide (K_2_O)	2.87
Magnesium Oxide (MgO)	1.82
Titanium Dioxide (TiO_2_)	0.89
Sodium Oxide (Na_2_O)	0.64
Dichlorine Monoxide (Cl_2_O)	0.16

**Table 3:** Mix proportions of the studied mortars [23].

Mix	Mix Proportion (g)							
	Dredged Sediment		OPC	GGBFS	MK	NaOH	Na_2_SiO_3_	Supplementary Water
	Solid	Water						
OPC	871.1	261.3	87.1	-	-	-	-	90.0
GSB	880.0	264.0	-	-	-	14.0	153.0	10.0
GSB_OPC	816.0	244.8	81.6	-	-	14.0	153.0	35.0
GSB_GGBFS	816.0	244.8	-	81.6	-	14.0	153.0	35.0
GSB_MK	816.0	244.8	-	-	81.6	14.0	153.0	35.0

**Table 4:** Molar ratios of the studied binders [23].

Molar Ratios	GSB	GSB_OPC	GSB_GGBFS	GSB_MK	Minimum Value	Maximum Value
SiO_2_/Al_2_O_3_	5.54	5.62	5.58	5.33	3.50	4.50
Na_2_O/Al_2_O_3_	0.57	0.5	0.53	0.48	0.80	1.25
H_2_O/Na_2_O	14.56	14.48	14.37	14.18	10.00	15.00
SiO_2_/Na_2_O	1.20	1.20	1.20	1.20	1.25	1.95

**Table 5:** Total porosity and pore repartition according to the classes defined by Anderson and Pratt [23].

Mix	Micropores (%)	Mesopores (%)	Macropores (%)	Porosity (%)
GSB	4.0	75.0	21.0	22.5
GSB_OPC	7.0	72.0	21.0	51.1
GSB_GGBFS	13.0	84.0	3.0	42.5
GSB_MK	27.0	65.0	8.0	39.4

**Table 6:** Relative mass loss related to each phase.

Mix	Total Mass Loss (%)	Mass Loss Related to Each Phase			
		Free Water	C-A-S-H	Geopolymer	Carbonates
GSB	13.92%	3.19%	-	5.74%	4.99%
GSB_OPC	15.65%	2.40%	11.95%	-	1.30%
GSB_GGBFS	15.07%	4.11%	9.82%	-	1.14%
GSB_MK	15.09%	3.56%	-	9.54%	1.99%

## Data Availability

The original contributions presented in this study are included in the article. Further inquiries can be directed to the corresponding author.

## Abbreviations

The following abbreviations are used in this manuscript:

FA	Fly Ash
GGBFS	Ground-Granulated Blast Furnace Slag
MK	Metakaolin
NMR	Nuclear Magnetic Resonance Spectroscopy
TGA	Thermogravimetric Analysis
PAU	Pauillac sediments
NaOH	Sodium Hydroxide
Na_2_SiO_3_	Sodium Silicate
EDS	Energy-Dispersive Spectroscopy
SEM	Scanning Electron Microscopy
Si	Silica
Al	Alumina
XRD	X-ray Diffraction
GPMB	Grand Port Maritime de Bordeaux
MBV	Methylene Blue Value
MIP	Mercury Intrusion Porosimetry
N-A-S-H	Sodium Aluminosilicate Hydrate
N-(C)-A-S-H	Calcium-Modified Sodium Aluminosilicate Hydrate
C-S-H	Calcium Silicate Hydrate
C-A-S-H	Aluminum-Substituted Calcium Silicate Hydrate
